# Association among house infestation index, dengue incidence, and sociodemographic indicators: surveillance using geographic information system

**DOI:** 10.1186/s12889-015-2097-3

**Published:** 2015-08-05

**Authors:** Waldemir Paixão Vargas, Hélia Kawa, Paulo Chagastelles Sabroza, Valdenir Bandeira Soares, Nildimar Alves Honório, Andréa Sobral de Almeida

**Affiliations:** Departamento de Endemias Samuel Pessoa, Escola Nacional de Saúde Pública, Fundação Oswaldo Cruz, Rua Leopoldo Bulhões, 1480, 6° andar, Manguinhos, CEP 21041-210 Rio de Janeiro, RJ Brazil; Departamento de Epidemiologia e Bioestatística, Instituto de Saúde da Comunidade, Universidade Federal Fluminense, Rua Marquês do Paraná, 303, 3° andar, Prédio Anexo ao HUAP, CEP 24030-210 Centro, Niterói, RJ Brazil; Núcleo de Apoio as Pesquisas em Vetores, Instituto Oswaldo Cruz, Avenida Brasil, 4365 Manguinhos, CEP 21045-900 Rio de Janeiro, RJ Brazil

**Keywords:** Dengue, Infestation index, Geographic Information Systems, Epidemiological surveillance

## Abstract

**Background:**

We identified dengue transmission areas by using the Geographic Information Systems located at local surveillance units of the Itaboraí municipality in state of Rio de Janeiro. We considered the association among the house infestation index, the disease incidence, and sociodemographic indicators during a prominent dengue outbreak in 2007 and 2008.

**Methods:**

In this ecological study, the Local Surveillance Units (UVLs) of the municipality were used as spatial pattern units. For the house analysis, we used the period of higher vector density that occurred previous to the larger magnitude epidemic range of dengue cases. The average dengue incidence rates calculated in this epidemic range were smoothed using the Bayesian method. The associations among the House Infestation Index (HI), the Bayesian rate of the average dengue incidence, and the sociodemographic indicators were evaluated using a Pearson’s correlation coefficient. The areas that were at a higher risk of dengue occurrence were detected using a kernel density estimation with the kernel quartic function.

**Results:**

The dengue transmission pattern in Itaboraí showed that the increase in the vector density preceded the increase in incidence. The HI was positively correlated to the Bayesian dengue incidence rate (*r* = 0.641; *p* = 0.01). The higher risk areas were those that were close to the main highways. In the Kernel density estimation analysis, we observed that the regions that were at a higher risk of dengue were those that were located in the UVLs and had the highest population densities; these locations were typically located along major highways. Four nuclei were identified as epicenters of high risk.

**Conclusions:**

The spatial analysis units used in this research, i.e., UVLs, served as a methodological resource for examining the compatibility of different information sources concerning the disease, the vector indices, and the municipal sociodemographic aspects and were arranged in distinct cartographic bases. Dengue is a multi-scale geographic phenomenon, and using the UVLs as analysis units made it possible to differentiate the dengue occurrence throughout the municipality. The methodological approach used in this research helped improve the Itaboraí municipality monitoring activities and the local territorial monitoring in other municipalities that are affected by this public health issue.

## Background

Dengue is the most important arbovirus that affects humans. It is transmitted by the sting of infected *Ae. aegypti* females. This species exhibits endophilic and anthropophilic behavior [1–3] and has ample distribution in urban and suburban environments, where there is a high population density. According to the World Health Organization, dengue puts approximately 2.5 – 3 billion people in more than 100 endemic countries at risk, especially in the Tropical and Subtropical areas [4]. Over the last two decades, dengue incidence has significantly increased in endemic areas, particularly in the Americas, where there is a co-circulation of four serotypes [5]. In Brazil, the number of notified cases between 2007 and 2012 was 3,730.507, and the Rio de Janeiro State and the Itaboraí municipality registered 628,708 and 16,383 cases, respectively [6].

Dengue transmission is essentially urban because this specific environment harbors all of the fundamental factors for its occurrence: humans, the virus, the vector and, most importantly, the political, economic, and cultural conditions that are favorable to the establishment of the transmission chain [1, 7]. Several combined elements can produce epidemiologic conditions for dengue virus transmission, including serotype circulation, higher human-vector contact, rapid population growth, rural–urban migration, inadequate basic urban infrastructure, and an increase of solid residue; these factors favor breeding availability in urban areas and subsequently increase *Ae. Aegypti* infestation [8–10].

The control strategies that are currently used include municipal sanitary surveillance to support surveillance and vector control actions, conducting entomological survey indicators, and monitoring the activity of the resistance of *Aedes* to insecticides using traps, but they have not been able to contain the endemic-epidemic process of the disease, which has reached the greater part of Brazilian municipalities situated in urban areas [11]. In this context, entomologic surveillance is a fundamental instrument for the evaluation and operationalization of the control program indicators for this arbovirus [12]. One of the challenges, however, is detecting the trustworthy entomologic indicators, which can estimate and correlate the density levels of *Ae. Aegypti* with dengue occurrence in a determined population [13].

The entomologic indicators that are traditionally used to monitor the populations of *Ae. Aegypti* are based on the presence and/or absence of immature forms of *Ae. Aegypti*. Among these, the house infestation index (HI) and the Breteau index (BI) stand out as approximate measures for dengue transmission risk. However, it is not always possible to observe strong associations with the incidence of dengue fever, which is possibly due to the inadequate quality of the entomological data collection and measures of vector infestation as well as the incidence data, which are based primarily on clinical diagnostics of asymptomatic or subclinical infections [1, 14].

Another aspect that is emphasized in the epidemiologic surveillance of dengue is the geographic scale that is used in control strategies for and research into the disease. The transmission dynamic is strongly related to the local environmental characteristics, which makes it possible to identify differences in the spatial and temporal distributions of the disease [15, 16]. The transmission dynamic is also related to the climatic conditions that are favorable for the production of *Ae. aegypti* [17], such as precipitation and temperature [18].

The Itaboraí municipality has had dengue epidemics since 2001, with a progressive infestation and dispersion of *Aedes aegypti* in many areas of the city. Therefore, the present article’s objectives are to use a Geographic Information System (GIS) at local surveillance units to identify transmission areas of dengue by studying the association among the house infestation index (HI), the incidence of the disease, and the sociodemographic indicators.

## Methods

### Area of study

**Fig. 1:**
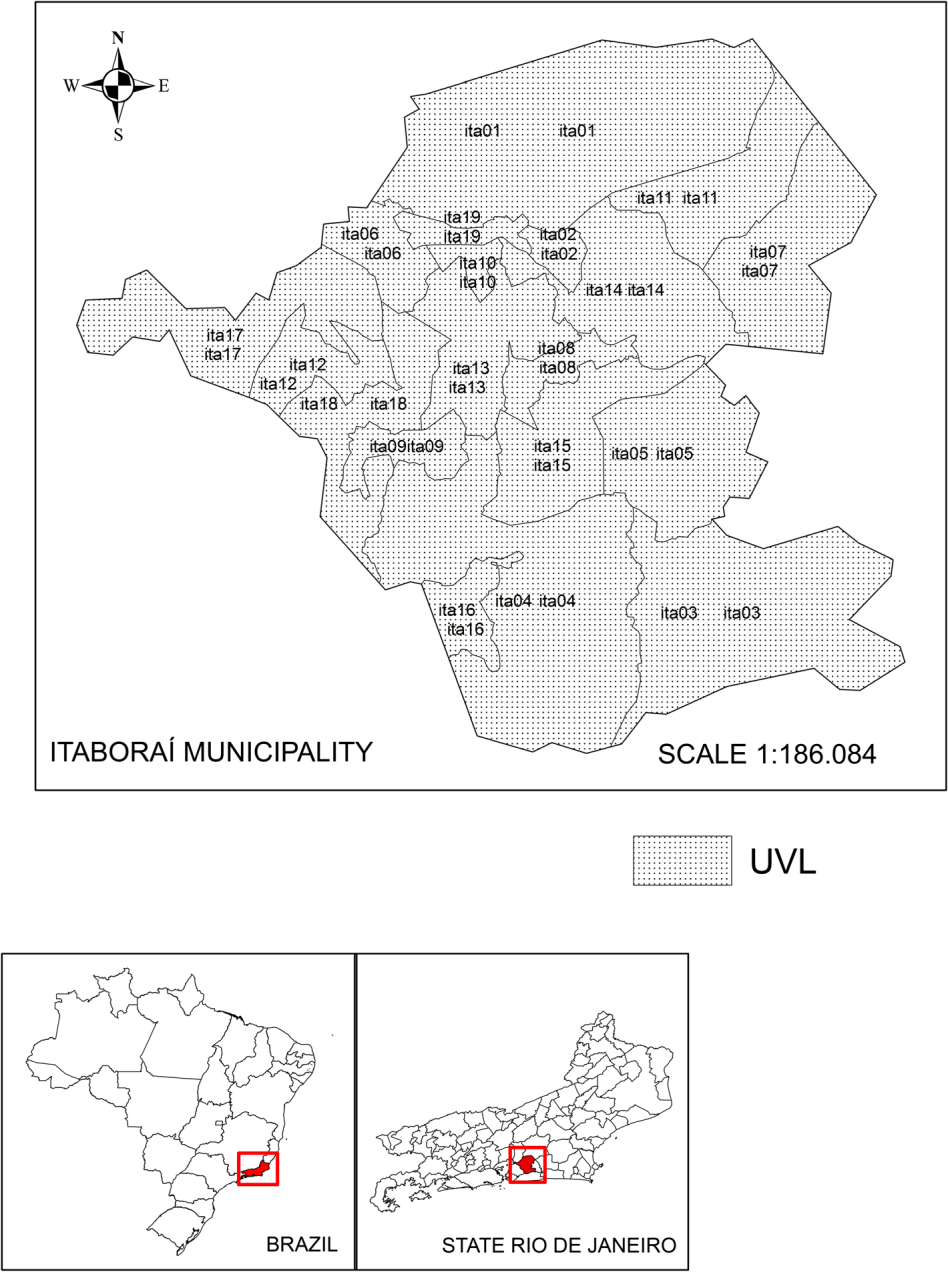
Map of the location of UVLs in the municipality of Itaboraí, State of Rio de Janeiro, Brazil

The Itaboraí municipality (latitude 22° 44’ 51” South, longitude 42° 51’ 21” West) is situated in the metropolitan region of Rio de Janeiro and has an area of 430.373 km^2^, which corresponds to 8.08 % of the region (Fig. 1). The population is 218,008 inhabitants, and the demographic density is 506.56/km^2^. Itaboraí is 17 m above sea level and is located 40 km from the municipality of Rio de Janeiro and 75,57 Km from the coastline and the Eastern region of the state. It has 79 neighborhoods (Ibge 2010) that are divided into 8 districts: Centro (thirty-three neighborhoods), Porto de Caixas (two neighborhoods), Itambi (eight neighborhoods), Sambaetiba (six neighborhoods), Visconde de Itaboraí (seven neighborhoods), Cabuçu (seven neighborhoods), Manilha (thirteen neighborhoods), and Pacheco (three neighborhoods). The districts of Cabuçu and Pachecos are predominantly rural with small urban nuclei and are characterized as rural–urban areas.

It is important to remember that the Itaboraí municipality is home to a large-scale industrial enterprise, the Petrochemical Complex of Rio de Janeiro (Comperj), which produces petroleum derivatives and petrochemical products. Its construction has brought about profound economic, social, and environmental changes throughout the Eastern region of the State [19].

### Study design

This is an ecological research study in which we utilized dengue cases that were identified by the National Disease Surveillance Data System (SINAN) and data regarding home infestation (Home Infestation Index and Breteau Index), which are available on the Yellow Fever and Dengue Information System (SISFAD). Variables regarding urban infrastructure, education, and demographic conditions, which were obtained from the 2010 Demographic Census of IBGE, were used to elaborate the sociodemographic indicators during the period from 2007 to 2008.

The SINAN is a system that contains the records of notifiable diseases from throughout the national territory, including suspected cases of dengue that are later confirmed by laboratory and/or epidemiological criteria. A suspected case of dengue constitutes a “*person who lives or has traveled in the last 14 days to the area where dengue is being transmitted or where the presence of Ae. Aegypti, which features fever, usually between 2 and 7 days and present two or more of the following manifestations: nausea, vomiting, rash, myalgia, arthralgia, headache, retro-orbital pain, petechiae or positive tourniquet test and leukopenia*” [6].

SISFAD is a system that allows for the computerization of data related to the control activities for the dengue vector of the National Dengue Control Program (PNCD). It has been in place since 1997. This system registers entomologic surveillance activities, which allows us to evaluate the effectiveness of the vector control programs [20].

### Entomologic data

The Home Infestation Index (HI) is the relationship, expressed as a percentage, between the number of positive homes and the number of homes researched. The HI was calculated to evaluate the infestation indicators of *Ae. Aegypti.* Health agents evaluate the vector infestation indices in the municipality five to six times per year.

To analyze the risk associated with the HI, we used the Health Department recommended classification as follows: Low-risk or satisfactory (HI < 1 %); Mid-risk or alert (HI <1–3.9 %); and high-risk or dengue outbreak (HI > 3.9 %). The HI was calculated for the entire research period, but for our analysis, we used the period from October 2007-March 2008. This was the interval in which the highest *Ae. aegypti* density occurred, which preceded the epidemic in 2008. Due to irregularities in monitoring, which resulted in a low completion rate of the Breteau Index (BI), we opted to exclude this index from the analysis.

The pending rate was also analyzed. The pending rate is the number of pending closed houses/buildings, abandoned properties, and locations where there was a refusal to inspect the endemic control agents (ECA). In addition, commercial properties that were considered unsuitable from a health point of view and that were not inspected by the agents were included. It is noteworthy that the outstanding properties are a major problem in combating viral vectors due to the lack of information about the existence of potential breeding sites. According to the National Health Department Guidelines for Prevention and Control of Dengue Epidemics, a high rate is considered to be 10 to 20 % of pending cases and is grave when the pending ratio is above 20 %. This represents a critical situation in which *Ae. aegypti* control requires urgent measures for to reduce its ratio.

### Epidemiological data

A total of 4,281 notified cases of dengue in the Itaboraí municipality were analyzed. Among them, there were 1,079 and 3,202 cases in 2007 and 2008, respectively. The monthly dengue incidence rates were also calculated for the years 2007 and 2008, as was the mean incidence of the high-magnitude epidemic for the period from January to August of 2008.

### Sociodemographic data

For the sociodemographic analysis, we used the variables that were available in the 2010 Demographic Census: the proportion of permanent private houses with piped water supply (HOUSEWATER), the proportion of permanent private houses with garbage collection (HOUSEGARBA), the ratio of male and female residents (RSEX), the proportion of permanent private houses with water supply from a well or spring on the property (HOUSEWATERW), the proportion of permanent private houses with other forms of water supply (HOUSEWATEROFORM), the proportion of permanent private houses with garbage burned on the property (HOUSEGARBABURN), the proportion of literate men (LITERAMEN), and the proportion of literate women (LITERAWOMEN).

### Geographic data

To analyze the dengue characteristics at the intra-municipal level, it was first necessary to make the many data banks that were used compatible to one territorial unit, which would allow us to overlap the information and compare the researched periods.

Using the information from the IBGE Census tracts (CTs) from the Department of Planning and Coordination of Itaboraí and from SISFAD sketches that referred to the Itaboraí municipality, we built a territorial basis in which we considered 19 Local Surveillance Units, or UVLs (Fig. 1).

A Local Surveillance Unit [21] is an area that is defined by operational criteria and has an adequate size to obtain epidemiologic, socio-economic, demographic, and other indicators, which allow for surveillance activities to be conducted in a municipality. In general, it is composed of an aggregation of the CT and is not necessarily an administrative unit; it is instead a neighborhood or a small group of neighborhoods.

The population estimates of the UVLs were calculated from the 2000 and 2010 Demographic Census, from the Family Health Program (FHP) data, and from SISFAD.

**Fig. 2:**
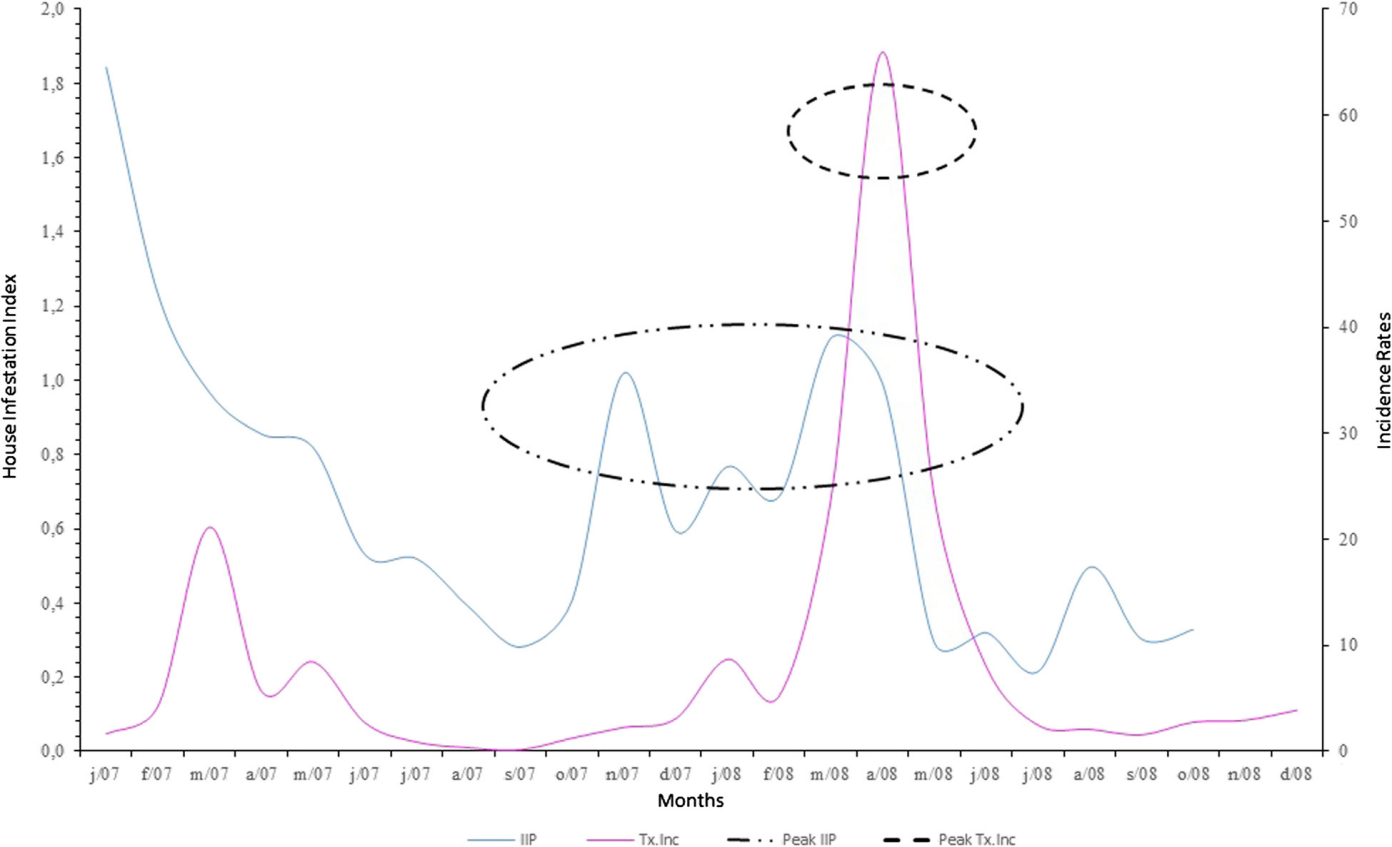
Monthly distribution of the dengue incidence rate and the house infestation index in 2007 and 2008, Itaboraí municipality, Rio de Janeiro State, Brazil

The UVLs were georeferenced using the Professional MapInfo 10.0 software, in which geographic coordinates were attributed to the control points created by the sketches. These control points were captured from Google Earth 6.0, from which we also obtained a convertor to transform the plain geographical coordinates – Universal Transverse Mercator (UTM), datum SAD 69, zone 23, south hemisphere [22]. The spatial stratification methodology proposed in this research for the Local Surveillance Units (UVL) in Itaboraí was done not only to make diverse data bases compatible but also to make the production and control processes for transmissible diseases, such as dengue, occur locally. The Local Surveillance Units, or UVLs (Fig. 2a), are mostly urban. UVL 01 has rural/industrial characteristics, and the Comperj is located there. UVLs 03, 04, and 07 have rural characteristics but urban (rural/urban) nuclei. UVLs 02, 05, 06, 08, 09, 10, 11, 12, 13, 14, 15 and 16, 17, 18 and 19 are considered urban.

### Data analysis

A descriptive analysis was completed for the entomological and epidemiological data of the municipality and its UVLs during the period studied. First, the quantitative data were analyzed regarding the notified cases, the dengue incidence, the HI, and the BI in both 2007 and 2008 in this municipality.

For each UVL, we calculated the mean incidence ratio of dengue by dividing the new case numbers registered in the period from January to August 2008 by the estimate resident population during 2008. To minimize the instability problem of the incidence rate calculated for small areas, we used the local empirical Bayesian approach [23, 24]. This approach includes spatial proximity effects by using the information from the regions that neighbor the geographical area to estimate the incidence rate. Empirical Bayesian procedures yield more reliable estimates because they use information from other areas to estimate the rates in a given region. In general, this procedure produces a set of incidence rates that, when they are presented on a thematic map, yield a less heterogeneous visual appearance than that produced by uncorrected incidence rates. Therefore, this pattern is usually referred to as smoothed.

Spatial distribution thematic maps of the local Bayesian incidence rate were built for the notified dengue cases (smoothed incidence rate) that corresponded to the higher magnitude range of the period from January to August 2008. Spatial distribution thematic maps of the HI, corresponding to the period from October 2007 to March 2008 and of the sociodemographic indicators from the 2010 Demographic Census [25] were also built.

We also employed napierian logarithm transformations (Ln) for the HI, the dengue Bayesian local incidence rate, and the sociodemographic indicators. A correlation matrix was built for the Pearson’s Coefficient among the HI, the smoothed dengue incidence rate, and the sociodemographic indicators. The software application used for this analysis was Statistica 6 [26]. The higher risk areas for dengue occurrence were detected using the kernel density estimate with the kernel quartic function. The flattening degree was controlled by means of the bandwidth, with a radius of 3,000 m [27]. The application used for the spatial analysis and the thematic maps was ArcGIS 10.0.

## Results

The monthly distribution of the HI of *Ae. Aegyptie* and the dengue incidence rates in 2007 and 2008 in the Itaboraí municipality are presented in Fig. 1. In 2007, the house infestation indices varied from 0.28 % to 1.84 %, and in 2008, they varied from 0.21 % to 1.11 %. In 2007, smaller HI values were detected during the coldest months of the year: June (0.53 %), July (0.52 %), August (0.39 %), and September (0.28 %). During the subsequent months, November, December, January, and February, the indices varied from 0.59 to 1.02 %. However, in March and April, these numbers increased to 1.11 % and 0.99 %, respectively (Fig. 2).

**Fig. 3:**
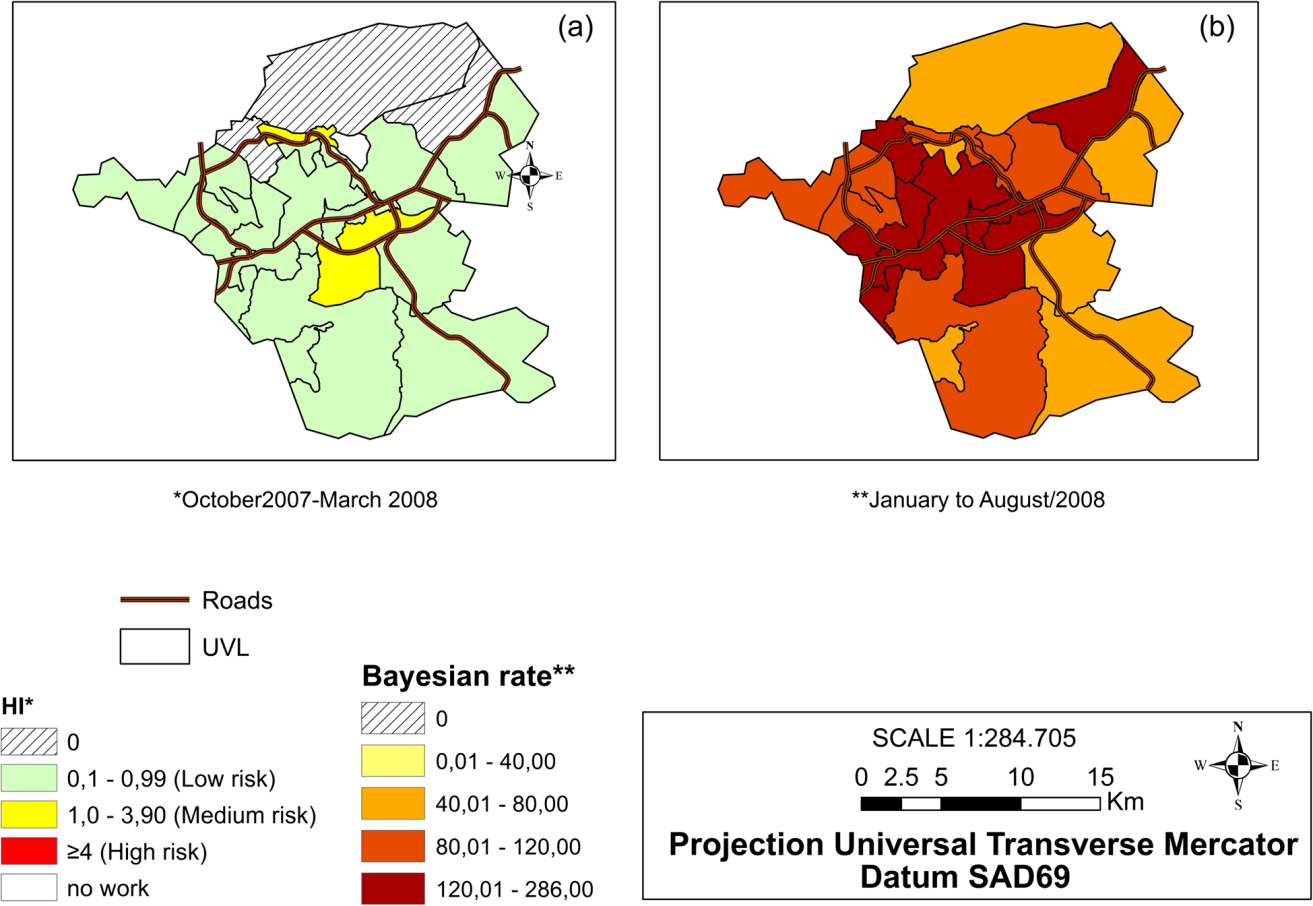
Maps of the house infestation index (**a**), and Bayesian dengue incidence rate (**b**), Itaboraí municipality, Rio de Janeiro State, Brazil

In Fig. 3, we can observe that the dengue incidence rates increased in the months of February and March, but the differences were small . In April 2008, however, a peak (65.94 per ten thousand inhabitants) can be observed in the incidence rate during the researched period, which was preceded by increases in the house infestation indices during November 2007 (1.02 %), decreasing between December and February, and increasing again in March and April 2008 (0.99 %).

The gross incidence rates in the Itaboraí municipality group in 2007 and 2008 were 52.54 and 154.46 cases, respectively, per 10 thousand inhabitants (data not presented).

It is important to highlight that the pending rates (closed houses or refusals) for the house infestation during this period were very high, reaching 21.85 % in 2007 and 19.21 % in 2008.

Table 1 contains the cases, incidence, and vector infestation indices (HI and BI) in the UVLs of the municipality. The UVLs (ita 03, ita06, ita08, ita09, ita11, ita12, ita13, ita14, ita15 and ita18) that had dengue incidence rates in 2008 greater than 100 cases per 10 thousand inhabitants are primarily urban, with the exception of UVL Ita03. Among the nine urban UVLs that had a high incidence, three had an HI above 1 %, two were close to this number, and the others had values less than 0.55 %. Although UVLs ita04, ita05, and ita17 did not have rates greater than 100 cases per 10 thousand inhabitants, they had an HI above 1 % in 2007.

**Table 1:** Distribution of cases and the House Infestation Indices (HI) and Breteau Indices (BI), the population density (Km^2^), and dengue incidence rates in 2007 and 2008, according to the UVLs of the Itaboraí municipality

Code_UVL	Area	Cases		HI		BI		Population density		Incidence^b^	
		2007	2008	2007	2008	2007	2008	2007	2008	2007	2008
ita01	rural/industrial	0	0	0.56	0	0.56	0	11.36	11.35	0.00	0.00
ita02	urban	0	0	^a^	^a^	^a^	^a^	144.77	144.48	0.00	0.00
ita03	rural	10	34	0.09	0.07	0.09	0.07	64.77	64.69	30.07	99.34
ita04	rural	18	85	1.3	0.87	1.3	0.87	147.02	150.77	6.18	32.14
ita05	urban	7	29	1.76	1.4	1.83	1.4	153.77	159.82	17.78	70.85
ita06	urban	17	105	0.04	0.16	0.04	0.16	447.70	470.72	41.64	244.58
ita07	rural	0	12	0.16	0.04	0.16	0.04	108.09	110.08	0.00	0.00
ita08	urban	291	551	1.04	0.8	1.18	0.8	3066.51	3053.52	156.67	321.34
ita09	urban	51	255	0.38	0.91	0.38	0.91	1843.09	1853.73	40.47	219.06
ita10	urban	12	5	0.37	0.23	0.37	0.23	603.86	595.44	17.43	29.46
ita11	urban	18	74	0.52	0.04	0.52	0.04	118.89	118.73	76.66	358.21
ita12	urban	18	131	0.37	0.44	0.37	0.44	508.53	540.55	16.40	112.30
ita13	urban	115	325	0.99	0.81	0.99	0.81	1147.01	1172.65	47.27	151.98
ita14	urban	117	374	0.94	0.47	0.96	0.48	1082.10	1102.64	36.73	100.39
ita15	urban	162	445	1.39	2.21	1.46	2.21	1296.96	1314.05	57.06	152.20
ita16	urban	3	22	0.46	0.31	0.46	0.31	394.74	386.36	61.17	294.12
ita17	urban	39	84	1.44	1.1	1.44	1.1	357.38	353.60	40.90	89.04
ita18	urban	180	609	1.2	0.83	1.22	0.83	1746.32	1749.57	53.68	170.47
ita19	urban	21	62	0.91	0.7	0.91	0.7	1779.05	1889.28	46.26	89.76

Source: State Department of Health (SES)

^a^UVL without work

^b^ For 10 000 inhabitants

Figure 3 shows the mean dengue incidence rates during the January to August 2008 epidemic period and the HI during its peak period (October 2007 to March 2008) immediately prior to the epidemic period. Most of the UVLs’ HI were below 0.99 % and were considered low risk. Although UVLs ita15 and ita19 (which were completely urban) had the highest HI (which was situated in the mid-risk range) in the time frame researched, the UVLs had dengue incidence indices of 80 to 120 notified dengue cases per 10 thousand inhabitants and 40 to 80 cases per 10 thousand inhabitants, respectively.

Furthermore, UVL ita02 (with a population density of 144.48 km^2^ in 2008) did not register the infestation index in the analyzed period because no local agents were working in this area. However, the Bayesian dengue incidence rate was over 80 cases per 10 thousand inhabitants. UVLs ita01 (rural/industrial), ita06, and ita11 (urban with population density of 470.72 and 118.73/Km^2^, respectively) had an HI equal to zero because the last incidence rates were greater than 120 cases per 10 thousand inhabitants and thus occupied the highest class distribution interval.

**Fig. 4:**
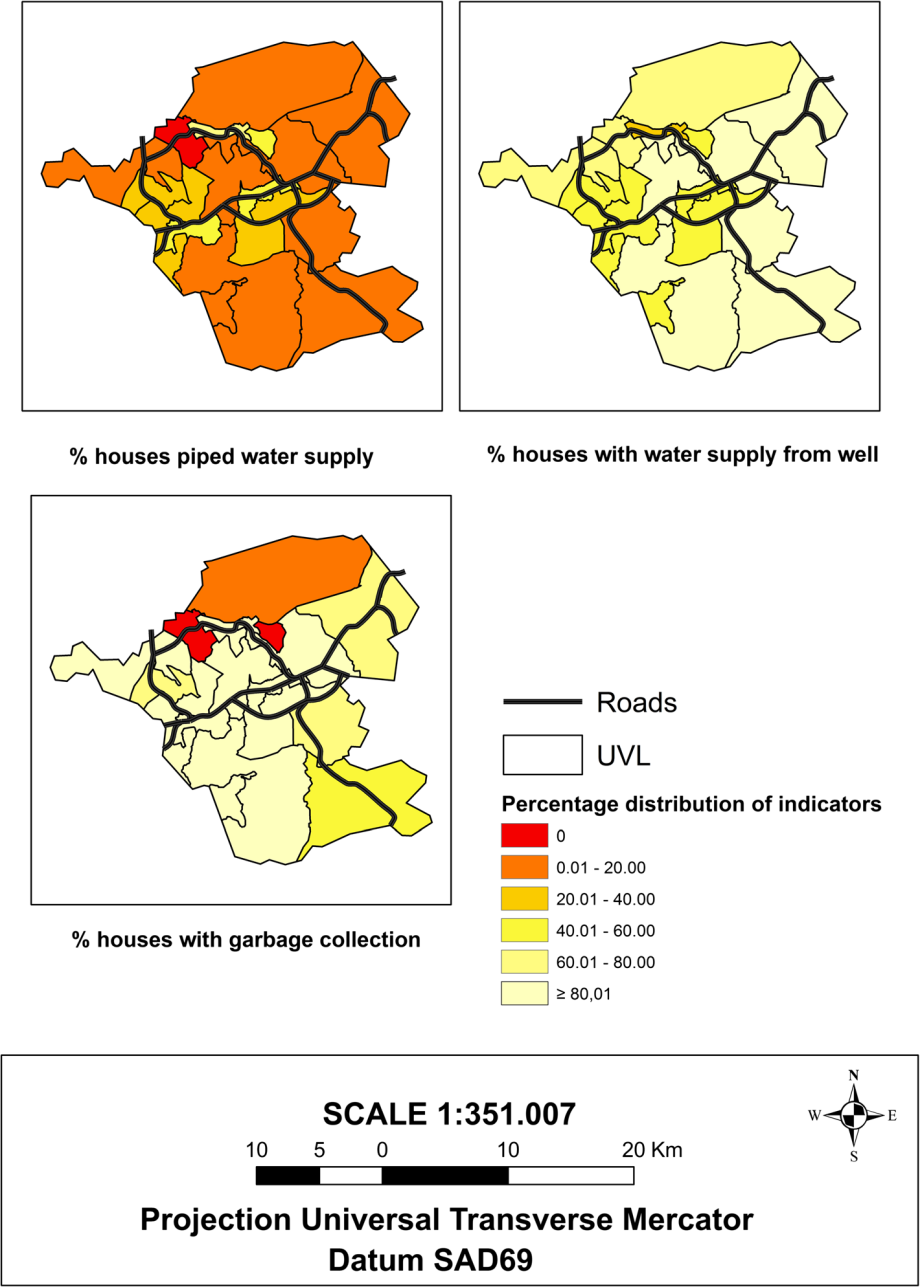
Maps of the sociodemographic indicators related to the property water supply (piped and well) and garbage disposal, which characterize the urban infrastructure, 2010 Demographic Census, Itaboraí municipality, Rio de Janeiro State, Brazil

By mapping the water supplies according to the UVLs, we can observe the precarious water supply on an intra-municipal scale where most UVLs, in which houses are linked to the piped supply, do not surpass the second interval of the class distribution (0.01–20.0). However, almost all of these UVLs contain houses that use wells as their water supply (Fig. 4). Considering the municipality as a whole, it can be observed that only 27.9 % of the properties are linked to the piped water supply and that approximately 80 % of the houses use a well to obtain their water.

Regarding the garbage collection in the house indicator, it can be observed that among the 19 UVLs, only 3 (ita01, ita02, and ita06), which are located in the north region, did not present this type of service for the property (Fig. 4).

**Fig. 5:**
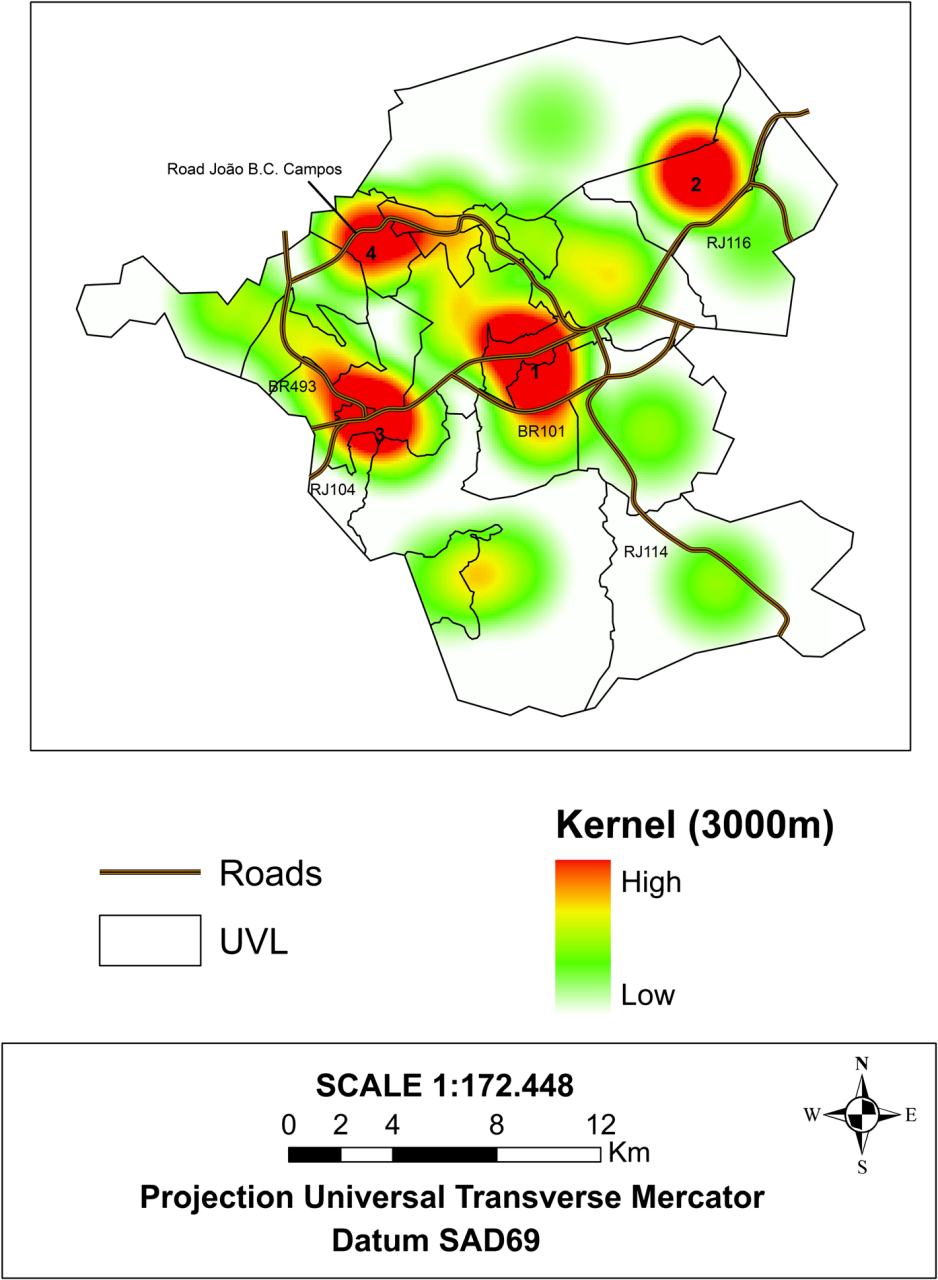
Map of the Kernel estimator of the dengue Bayesian rate (epidemic range of 2008) and the main highways, Itaboraí municipality, Rio de Janeiro State, Brazil

In the Kernel estimate analysis, we observed that the highest dengue risk regions were located in the UVLs that had the highest population densities; these regions also happen to be located along major highways. Four nuclei were identified as high risk. The first nucleus is a large central nucleus composed of three UVLs (ita08, ita13, and ita15); is located along highway BR 101; has a concentrated population of 72,439 inhabitants, which represents 34 % of the population of the municipality in 2008 (211,697 inhabitants); and has a demographic density that varied from 11,172.65 to 3,053.52/ Km^2^. The second nucleus (Nucleus 2), which is located to the right of the first, is in the northeast region of the municipality along state highway RJ 116 and is composed of three UVLs, one rural (ita07) and two urban (ita11 and ita14). These three units together contain 34,900 inhabitants, approximately 17 % of the total population, and have a demographic density between 110.08 and 1,102.64/ Km^2^. The third nucleus (Nucleus 3) is located left of the first nucleus; is composed of two UVLs, with a high population density (ita09 and ita18); and is close to highways BR 101, BR493, and RJ 104. The total population is 48,325 inhabitants, which represents 23 % of the population of the city. The fourth nucleus (Nucleus 4), which is of lesser intensity, is composed of the urban UVL ita06, is located on the border of the highway João Batista Caffara Campos, and has a population of 4,293 inhabitants and a demographic density of 470.72/Km^2^ (Fig. 5).

Table 2 presents the correlation results obtained for the HI, the dengue Bayesian incidence rate, and the sociodemographic indicators. The HI is positively correlated with the local Bayesian dengue incidence rate (*r* = 0.641/*p* = 0.01). Of the eight sociodemographic indicators used in this research, four were statistically significant compared with the HI, and two were significant compared with the local Bayesian dengue incidence rate (INCBAYES). The proportion of properties with piped water (HOUSEWATER), those with garbage collection (HOUSEGARBA), and the sex ratio (RSEX) positively correlate with HI; for the first two, p < 0.05, and for the last, p < 0.01. Garbage collection is positively correlated with dengue incidence (*p* < 0.05).

**Table 2:** Correlation matrix of HI^1^ (October 2007 to March 2008), Bayesian incidence rate^a^ (January to August 2008), and sociodemographic indicators^a^ – Pearson’s Coefficient. Itaboraí municipality, Rio de Janeiro State, Brazil

	HI	Incbayes	% Housewater	% Housegarba	RSEX	% Housewaterw
HI	1.00					
*p* value						
INCBAYES	0.641**	1.00				
*p* value	0.010					
% HOUSEWATER	0.611*	0.411	1.00			
*p* value	0.015	0.090				
% HOUSEGARBA	0.635*	0.515*	0.143	1.00		
*p* value	0.011	0.035	0.584			
RSEX	0.679**	−0.216	0.504*	−0.740**	1.00	
*p* value	0.005	0.374	0.033	0.001		
% HOUSEWATERW	−0.475	−0.310	−0.810**	−0.282	0.467*	1.00
*p* value	0.074	0.196	0.000	0.273	0.044	

^a^IIndicators with neperian

transformation

*p < 0.05 **p < 0.01

## Discussion

In Brazil, dengue epidemiologic surveillance is performed according to the vector density levels of *Ae. aegypti* and the record of human cases of the disease. For each of these, there is a specific data system. SISFAD is used for the entomologic surveillance activities, and SINAN is used for notifications concerning infected individuals and those with a suspected infection.

The most broadly used index to estimate the vector density levels of *Ae. aegypti*, which is available in SISFAD, is the Breteau Index (BI). During the data bank elaboration, we observed a similarity between the BI and the HI in the analyzed Local Surveillance Units. However, because of the low quality and the incompleteness of this indicator for the Itaboraí municipality, it was not possible to use the BI.

Some entomologic indicators of *Ae. aegypti* are related to the immature stages or the adult form of the infecting females. Due to readiness and reproduction, the indicators that are based in larvae are the ones that health services use. It is important to consider that the pupa levels could be more appropriate because the goal is to estimate the productivity of emergent individuals and, in doing so, to monitor the transmission risk and operationalization of the control activities [28]. For this research, we used the HI, which resulted in low numbers of dengue transmission levels in the municipality compared with those announced by the control program. Nevertheless, although the rates were not as high as those observed in January 2007, in 2008, the rates stayed compatible with the disease transmission. Furthermore, a transmission pattern was observed in which the increase of the vector density preceded the increase of the disease’s incidence rate in the epidemic range from January to August 2008. This was possibly mediated by the number of circulating serotype-susceptible people [29, 30]. In Rio de Janeiro State in 2008, the dengue case incidence peak occurred during March and April3 [31], similar to the peak observed in Itaboraí.

In the same year, there was a large disease epidemic in the State that was characterized by high lethality and the presence of a significant number of cases in a grave stage of the disease. The predominant virus was DENV2 [32]. Favorable environments for the proliferation of the vector *Ae. aegypti* could be produced due to the low proportion of properties that are connected to the piped water supply in Itaboraí, which caused the population to create water storage alternatives in their homes for daily consumption [8, 15, 33, 34]. It is common, though the properties are connected to the piped water supply or even to garbage collection, for these services not to be regular, which forces the population to use improvised water reserves and makes them susceptible to dengue transmission [35, 36]. Using the Kernel estimate, we identified differences in the incidence rate among the territorial units analyzed. The highest incidence rates occurred in those units that were densely occupied and were located close to an urban nucleus or along a main municipality highway, similar to those verified in other studies in the municipalities close to the metropolitan region of Rio de Janeiro State [33, 37]. This statistical tool is valid for the health service because it can provide information to obtain relevant subsidies for the disease control activities [33, 38].

Regarding the correlation observed between the HI and the dengue Bayesian rate, the interval analyzed must be observed (January to August 2008) because it coincides with the reintroduction of the serotype 2 (DENV-2) in Rio de Janeiro State. When a virus that differs from those that are already circulating in the population is introduced, the presence of susceptible individuals can intensify the transmission process of the disease. Related to this fact is the increase in the *Ae. aegypti* population density that occurred due to the elevated temperature and humidity, which increases during summer and autumn [11, 18]. It is possible to emphasize in this study that the highest infestation rates were observed predominantly in the summer, which is the season that had the highest temperatures and periods of rain.

Another aspect is the presence of the petrochemical enterprise (Comperj) in the Itaboraí municipality, which began construction in 2006. The arrival of large population contingents, the increase in the circulation of susceptible individuals in areas of precarious basic sanitation conditions, the high population densities, and the increment in the population mobility all favor the possibility of vector density elevation and the increase in the vulnerability to the endemic in all of the areas that have been influenced by the industry. It is worth noting that the current vector control measures will persist. That is, if dengue transmission depends predominantly on population immunity, new epidemics will occur, as has occurred in other municipalities in the state of Rio de Janeiro, regardless of the presence of Comperj. However, the construction of this project certainly favored/amplified the occurrence of the 2007–2008 epidemic in Itaboraí. Therefore, it is important that continuous monitoring be conducted for subsidizing control actions and monitoring the disease in the city.

Associations were identified among the HI, the dengue incidence rate, and the sociodemographic indicators. We observed a positive association between the HI and the proportion of properties that were connected to the piped water supply. It is important to understand that only 27 % of the properties have access to this service. However, the proportion of properties with wells, which are a possible alternative to the lack of piped water, is not associated with either the HI or the dengue incidence rate. This finding may be related to the irregularity of this service [35, 36] because this population also uses contained water storage, which facilitates the presence of potential breeding sites in the home and its surroundings. In contrast, the proportion of properties with adequate garbage collection was positively associated with both of these variables.

The observed association between the RSEX and HI indicators suggests that the presence of man (people) is strongly associated with the observed values of the vector infestation index in UVLs. Although we do not show the result of the calculation of this indicator in the manuscript, it was observed that among the 19 UVLs in the municipality of Itaboraí, five (5) have more men than women, and 14 have more women, though in 13 of the latter, the difference is very small. It should also be noted that in seven (UVLs 8,9,13,14,15 18 and 19) of the 13 UVLs in which there was a small difference between the numbers of men and women, the population density was very high. This situation shows that the REX indicator can function as a proxy for population density in the study area.

The urban infrastructure indicators should not be interpreted as risk factors for the disease, which should instead be associated with the magnitude or the house infestation indices in a linear pattern. The indicators should be interpreted as indirect markers of the occupation process and of the urban soil use, which indicates areas with distinct conditions of endemic receptivity. Receptivity is the “set of environmental, social, and behavioral characteristics that allow the reproduction of parasites and their maintenance in communities” [39].

Because of the difficulty in identifying the preferred deposits of *Ae. aegypti* in the UVLs in Itaboraí in 2007 and 2008, it was not possible to include this information in the analysis. However, we observed that the higher proportions observed in the municipality occurred in medium and large deposits (barrel/cask/vat), corresponding to 34.12 % and 35.29 %, respectively. This information corroborates the previous findings for the municipal scale [28, 34, 40].

Furthermore, the spatial analysis units used in this research (UVL), which served as a methodological resource to determine the compatibility of different information sources on the disease, the vector indices, and the municipal sociodemographic aspects, were arranged in distinct cartographic bases. Dengue is a multi-scale geographic phenomenon, and using the UVLs as analysis units made it possible to differentiate the dengue occurrence rates in the municipality.

This research has some limitations. The data related to the disease cases were recorded by residence address in SINAN and not by the probable infection local. This might lead to an over-estimation of the epidemiologic indicators.

For the HI analysis, the number of pending cases (closed houses or refusals) was high, which gave the false impression that the entire area that was inspected was inspected by the municipal agents of endemic control. It is understood that 100 % of the properties must be treated, which has evident implications for the control strategies adopted in the municipality, because closed properties might have vector focuses that cannot be identified in time. Regarding the SISFAD, aside from the restrictions regarding data reliability, we believe that the information obtained will be important for analyzing the dengue transmission dynamics in Itaboraí.

## Conclusions

Although it has its limitations, the present research, which used Local Surveillance Units, allowed us to identify distinct transmission areas (susceptible) for the disease by using the information available in the Itaboraí municipality. Therefore, the methodological approach employed helped improve the monitoring activities in both the studied area and the local territorial surveillance performed in other municipalities that are affected by this public health problem.
